# A Novel Framework for Modeling Person-to-Person Transmission of Respiratory Diseases

**DOI:** 10.3390/v14071567

**Published:** 2022-07-19

**Authors:** Jason Rodriguez, Owen Price, Rachel Jennings, Amy Creel, Sarah Eaton, Jennifer Chesnutt, Gene McClellan, Sweta R. Batni

**Affiliations:** 1 Applied Research Associates, Inc. (ARA), 4300 San Mateo Blvd NE, Suite A220, Albuquerque, NM 87110, USA; jrodriguez@ara.com (J.R.); oprice@ara.com (O.P.); rljennings@ara.com (R.J.); acreel@ara.com (A.C.); sschmitt@ara.com (S.E.); jchesnutt@ara.com (J.C.); gmcclellan@ara.com (G.M.); 2 Defense Threat Reduction Agency (DTRA), 8725 John J. Kingman Road #6201, Fort Belvoir, VA 22060, USA

**Keywords:** disease transmission, COVID-19, SARS-CoV-2, transport and dispersion, infectivity, disease transmission, respiratory mechanics, respiratory virus modeling, sensitivity analysis

## Abstract

From the beginning of the COVID-19 pandemic, researchers assessed the impact of the disease in terms of loss of life, medical load, economic damage, and other key metrics of resiliency and consequence mitigation; these studies sought to parametrize the critical components of a disease transmission model and the resulting analyses were informative but often lacked critical parameters or a discussion of parameter sensitivities. Using SARS-CoV-2 as a case study, we present a robust modeling framework that considers disease transmissibility from the source through transport and dispersion and infectivity. The framework is designed to work across a range of particle sizes and estimate the generation rate, environmental fate, deposited dose, and infection, allowing for end-to-end analysis that can be transitioned to individual and population health models. In this paper, we perform sensitivity analysis on the model framework to demonstrate how it can be used to advance and prioritize research efforts by highlighting critical parameters for further analyses.

## 1. Introduction

Upon recognition of the novel infectious agent SARS-CoV-2 in 2019, laboratories across the world began to conduct fundamental research on the pathogen and the disease (COVID-19) to evaluate host transmissibility, pathogen survivability and transport, and severity and infectiousness to assess possible impacts. A 2020 Nature review showed the exponential increase in published papers related to SARS-CoV-2 and COVID-19, as well as the trends in topics [1]. The review demonstrated that research on epidemic modeling and controlling the spread of the disease initially outpaced research on diagnostics and testing, public health, and hospital mortality. Early, heavily cited research showed evidence of person-to-person transmission [2], clinical features of infected individuals [3], and transmission stemming from asymptomatic individuals [4]. As respiratory transmission became increasingly recognized as the dominant mechanism of contagion [5,6], modeling efforts pivoted towards better understanding incubation periods [7], aerosol survivability [8], and reproduction numbers [6,9,10]; these studies were geared towards understanding the spread of COVID-19 and mechanisms for mitigating casualties. Due to the unknown nature of the disease in these early days and no modern pandemic to act as a foundational case study, model parameters and considerations were missing or not well characterized/defined, which could have resulted in additional uncertainties around results. Studies have presented models of particle generation and transport and even considered the importance of particle size [9,10]; this paper adds to previous work by presenting a complete model framework of person-to-person disease spread by a respiratory transmission that couples particle generation, transport, and deposition to aid researchers, along with a methodology for understanding the influence of model parameters on results. Although this paper uses SARS-CoV-2 and COVID-19 as a case study for the model, the authors are presenting a generalized framework for communicable infectious respiratory pathogens. We are using the framework and the SARS-CoV-2 parameterization to perform a sensitivity analysis that aims to uncover the most influential parameters when considering person-to-person disease transmission.

Early models of SARS-CoV-2 transmissibility were limited, in that the characterization of risk was missing infectivity and resulting disease severity. The former is typically characterized by a dose-response function with a median effective dose (ID_50_) and a measure of spread (i.e., probit slope or confidence bounds) [11,12,13,14], whereas the latter involves models that characterize onset time, progression of illness, and outcomes with or without treatment. There were early attempts to derive an ID_50_ from transmission data by correlating it to a reproduction number [15]; however, a tissue culture ID_50_ (TCID_50_) in ferrets was not determined until later in the pandemic [16]. Similarly, the ID_50_ in non-human primates for both symptomatic/asymptomatic expressions of illness were determined nearly a year after the start of the pandemic [17]. Recently, an intranasal administration ID_50_ for humans was estimated to be approximately 10 TCID_50_ [18]. Measured values of ID_50_ vary significantly from species-to-species and introduce uncertainty in risk assessment. Other missing parameters from much of the early research that could further add uncertainty include:

1. Information on aerosol transport and survivability that accounts for particle size; larger particles may be less susceptible to environmental conditions [19] and smaller aerosols can remain suspended in the air well down-wind from an infectious person [20,21,22].
2. The relationship between RNA copies from PCR assays and live virions; estimates of this value differed by orders of magnitude [23,24]; however, understanding the viral load of respiratory droplets is crucial to quantifying the degree of transmissibility.
3. The viral content of exhaled particles generated as a function of the disease stage; a necessary component to understanding the window of communicability for infectious disease modeling, but also isolation requirements.

The degree to which these parameters are vital components of infection risk likely varies by disease but understanding prediction sensitivity to these parameters will result in the ability to assess individual parameter impacts and prioritize their derivation. Determining critical parameters would lead to comprehensive risk assessments where the generation and presentation of an exposure environment can be directly correlated to human health effects. An understanding of the missing or incomplete data ensures better quantification of uncertainty and presents best/worst case scenarios for infections and mortalities in risk assessment.

At a high level, the proposed framework captures important environmental and biological features that impact emission, transport, infectivity, and disease progression. The framework begins with an infectious individual emitting particles into an environment as they breathe, talk, or cough (particle generation). Virus-laden particles immediately degrade via evaporation with further decay determined by the ambient temperature, humidity, and sunlight, and larger particles settle due to gravity, removing them from the air (particle transport). The remaining particles present as an air concentration to a susceptible human. The number of inhaled particles is estimated across different deposition sites in the respiratory tract [25], which can either be taken individually or summed across deposition sites to calculate the likelihood of infection (human response); thus, the framework may be used to capture the transmission dynamics for a variety of contagious respiratory diseases.

Using SARS-CoV-2 as a proof-of-concept, this paper provides qualitative descriptions of the three submodels (i.e., particle generation, particle transport, and human response) that form the end-to-end workflow, highlighting essential features of each (Section 2.1, Section 2.2, Section 2.3). The baseline simulation scenario and approach for conducting a sensitivity analysis of the full system are then defined (Section 2.4 and Section 2.5). Results of the individual submodels, complete workflow, and sensitivity analysis are presented (Section 3). Derivations and detailed model descriptions as well as accompanying source code are provided in Appendix A, Appendix B, Appendix C: Appendix A contains equations and parameter descriptions for the models, Appendix B contains additional results from the sensitivity analysis, and Appendix C contains the Python implementation of the modeling framework.

## 2. Materials and Methods

**Figure 1:**
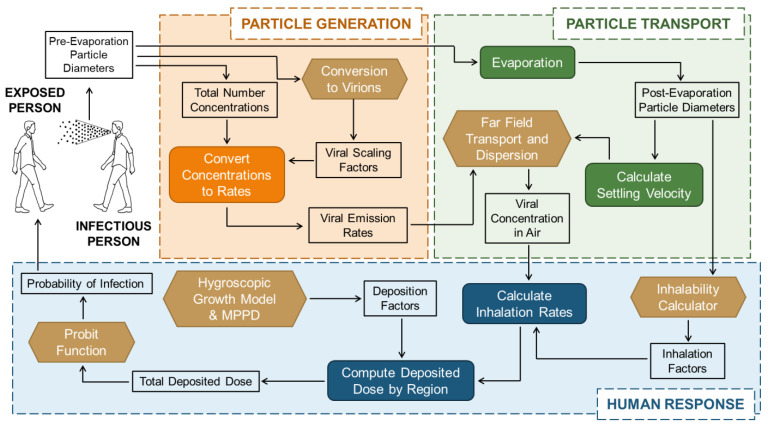
End-to-end workflow of the computational framework. The probability of infection of a susceptible person is expressed as a function of particle emission by an infectious person.

The model framework presented in this paper is designed to be used to analyze a respiratory disease pathway from particle generation through the transport and dispersion of the particles, ending with inhalation and infectivity. The workflow of this framework is summarized in Figure 1. Note that the figure uses the terms “Particle Generation” and “Particle Transport”. Many authors use the term “aerosol” to describe small particles, typically below five microns in diameter, and “droplet” for larger diameters that tend to settle more quickly; this difference in terminology, and the need to develop a single term for what has typically been classified as aerosols and droplets, has been discussed in the literature [26]; this paper does not distinguish between the two particle size groups as the authors are presenting a generalized framework using algorithms that account for the full range of sizes: a lower bound that is typically dictated by the size of a single infectious agent and an upper bound that is dictated by the limits of respiration (i.e., respirable and inhalable particle size). Throughout this paper, we use “particle” as the single term to describe emissions from the respiratory tract, regardless of size or emission mode.

For this paper, we will use SARS-CoV-2 as the basis for discussion, but the model framework is designed to be threat agnostic and includes generalized parameters for person-to-person spread of other contagious respiratory diseases transmitted via the respiratory tract. The first three subsections describe the submodels that form the computational workflow; a detailed treatment of each submodel is provided in Appendix A. The last two subsections provide the considerations and analysis underscoring the Results section.

### 2.1. Particle Generation

To determine the initial air concentration of virus-laden particles, we consider a *particle generation* submodel. The composition, rate, and initial size distribution of particles emitted by an infective person is dependent on the mechanism of emission and the viral load in the upper respiratory tract (URT)/lower respiratory tract (LRT) in the infectious person [27].

Particles are generated and emitted by healthy and diseased individuals. Respiratory particles produced by infected individuals may carry airborne pathogens, and the surface deposition and inhalation of these particles are the primary mechanisms by which many contagious diseases, including SARS-CoV-2, are transmitted [28]. There are three primary routes of exposure, in general, for respiratory particles from an infectious individual to a susceptible individual: (1) respiratory particle emission and fomite deposition on surfaces [29]; (2) respiratory particle emission and direct deposition to mucous membranes or inhalation in close contact situations [30,31]; and (3) respiratory particle emission and inhalation of the suspended airborne particles [32]; this paper and model framework focuses solely on the third route of exposure.

The particle generation model describes the number, size distribution, and composition of particles generated by an infectious human throughout the course of the disease. The purpose of this model is to estimate the emission rate, size distribution, and composition of the particles as they exit the infectious individual’s oral and nasal cavities. To determine this environment, the model first characterizes the individual’s viral load in the upper respiratory tract (URT) and lower respiratory tract (LRT) at the time of the emission event. Studies with SARS-CoV-2 have shown varying viral load as a function of time and stage of infection [33,34,35]. The mechanism of emission determines where particles originate [36]; the composition of those particles [37,38,39], including virion counts per particle as derived from viral load [40,41,42]; and the size and number of those particles [38,43,44].

### 2.2. Particle Transport

To determine the exposure particle concentration and size distribution required for an inhalability calculation, we consider a *particle transport* model. Concentration in the air is dependent on humidity, temperature, sunlight, particle composition, and the initial virus-laden particle emission rate [43]; these factors affect the number of particles present in a volume of air, the size of those particles, and the number of live virions in those particles.

The particle transport model characterizes the transport, dispersion, and fate of particles and their viral component in the absence of a host (i.e., the surrounding environment). The purpose of this model is to receive inputs from the particle generation model to estimate the particle (and, specifically, virion) concentration downwind from the infectious person. In indoor environments, air circulation, ventilation, and filtration are crucial components of characterizing the environment, with ultraviolet (UV) radiation and temperature likely playing a less significant role [44]. Humidity is important in indoor environments, particularly in considering the impact particle size due to drying has on filtration and settling. For indoor environments, box models such as CONTAM and FaTIMA [45,46] have been used to estimate concentrations over time; these models typically estimate the steady-state concentration over time, accounting for particle removal via settling, filtration, air exchange, or other factors. For outdoor environments, humidity, UV radiation, and temperature play a significant role in particle dehydration and particle survivability. For example, particles evaporate slower in humid environments, likely resulting in faster settling [47], UV radiation diminishes viral activity [43], and experiments have shown crystallization of particles can occur after a particle dehydrates, potentially leading to a decrease in infectivity [48].

### 2.3. Human Response

To calculate the output of an end-to-end model, we consider a *human response* model of symptomatic infection and severity of the disease. Severe cases drive resource requirements, resource shortfalls, and fatalities [49,50]. By considering infectivity and severity in a risk assessment, it becomes clearer which models and parameters are most critical across the calculation. Significant drivers of disease severity are demographics [51], comorbidities [52], and dosage [17]. Severity depends on whether an individual is infected, which is typically determined by an ID50 and probit slope [9]. ID50 and probit slope can change depending on the deposition site [53,54,55], which is contingent upon particle size; inhalability is also affected by particle size and wind speed [56,57].

Infectivity is only the first part of a human response model—disease severity and outcome are important to understanding impacts on human response. A viral disease that generally presents as mild-to-moderate across a large portion of the population, such as the common cold, will have significantly fewer impacts than a disease that generally presents as severe-to-fatal, such as smallpox. COVID-19 has been shown to present from mild to lethal at different distributions depending on the stage of the pandemic [58,59] and the variant [60].

### 2.4. Numerical Simulations

To illustrate the behaviors produced first by the submodels, and then by the complete workflow, the end-to-end model was parameterized to model the emissions, transport, and deposition of SARS-CoV-2. We chose a set of scenarios that could be used to illustrate the utility of the model and provide some insight into parameter influence on risk assessments. Simulations run for this effort consider variations of the following example scenario: suppose two individuals simultaneously enter a single, uncontaminated room with a floor surface area of 25 m^2^ ($A_{floor}$) and volume (V) of 100 m^3^. At the time of entry (t=0), one individual is assumed to be newly infectious and the other SARS-CoV-2 susceptible. The two individuals stand apart at a distance of greater than 2 m, as per social distancing guidance. In our simulations, the infectious person emits SARS-CoV-2-laden particles via breathing, speaking, and/or coughing immediately upon entry and continues to do so at a constant rate of $E_{i}$ for eight hours. We did not consider sneezing for these simulations, as sneezing was not a significant sign/symptom of the alpha variant [3,7,61]. Sneezing could be included in future analyses, particularly as we consider biological agents with a higher incidence of sneezing. We assumed the individual who is susceptible to SARS-CoV-2 spends six hours in the room; this decouples the emission time and exposure time while still providing enough simulation time to perform the sensitivity analysis. Thus, for a given particle diameter $d_{i}$, the corresponding viral emission function $G_{i}\left(t\right)$ is assumed to be piecewise constant such that:

$$G_{i}\left(t\right)=\{\begin{array}{ll} E_{i}, & t\in \left[0,8\right]\\ 0, & otherwise \end{array}$$

Far-field transport and dispersion dynamics of the time-dependent viral concentration in the room, $C_{vi}\left(t\right)$, are governed by a simplified form of the ordinary differential equation (ODE) used in FaTIMA to estimate concentration as a function of time [62]; this simplified ODE for $C_{vi}\left(t\right)$ is given by:

$$\frac{dC_{vi}\left(t\right)}{dt}=\frac{1}{V}G_{i}\left(t\right)-\left[\frac{1}{V}\left(Q_{r}+v_{si}A_{floor}\right)+k_{inf}\right]C_{vi}\left(t\right)$$

In this scenario, the infectious person is the only source of contamination, and virus is removed by three mechanisms: removal via ventilation, gravitational-induced settling on the room floor, and viral decay. No consideration is given to airflow from other sources outside the room (i.e., open window) and filtration (i.e., air purifier).

The room is assumed to be uncontaminated at t=0 (i.e., $C_{vi}\left(0\right)=0$). Using this initial condition along with the above representation for $G_{i}\left(t\right)$, it can be shown that $C_{vi}\left(t\right)$ satisfies:

$$C_{vi}\left(t\right)=\{\begin{array}{cc} \frac{E_{i}}{\hat{\alpha }V}\left[1-e^{-{\hat{\alpha }}_{i}\left(t\right)}\right], & 0\leq t\leq 8\\ \frac{E_{i}}{{\hat{\alpha }}_{i}V}\left[e^{-{\hat{\alpha }}_{i}\left(t-6\right)}-e^{-{\hat{\alpha }}_{i}\left(t\right)}\right], & t8 \end{array}$$

where ${\hat{\alpha }}_{i}=\frac{1}{V}\left(Q_{r}+v_{si}A_{floor}\right)+k_{inf}$.

The SARS-CoV-2 susceptible person is assumed to be continuously exposed for a total of six hours, starting at t=0. Table 1 summarizes the values assigned to parameters for this baseline scenario. Unless stated otherwise, parameter values are fixed at their baseline values. Numerical simulations were conducted for varying particle emission mechanisms by the infectious individual (e.g., breathing, speaking, and coughing). The computational framework, including all three models, was implemented in both MATLAB and Python in accordance with best practices for coding verification. The Python implementation is available in Appendix C. 

### 2.5. Sensitivity Analysis

Extended Fourier Amplitude Sensitivity Test (eFAST), a variance-based global sensitivity analysis method [59], is applied to the end-to-end computational framework. eFAST employs Fourier transformations to decompose output variance, which is then attributed to fluctuations in the varied parameters using Sobol’s first-order and total sensitivity indices [58,60,61]. eFAST is known to be computationally intensive, and therefore only a subset of parameters can be included in the analysis. In this paper, we focus on parameters whose estimates will presumably introduce the most uncertainty to model predictions, as they are poorly understood or most likely to be unknown in real life.

Parameters selected for sensitivity analysis include evaporation diameter ratio ($r_{evap}$), minute ventilation of the infectious individual ($\varepsilon_{dz}$), median viral load ($\mu_{VL}$), standard deviation of the viral load ($\sigma_{VL}$), viral decay rate ($k_{inf}$), super spreader emission factor (λ), infectivity ratio ($r_{inf}$), median infectious dose ($ID_{50}$), and slope of the probit function (β). Prior to analysis, distributions for each selected parameter must be defined [61]; the distributions assigned to each parameter are provided in Table 2. Note that for uniformly distributed parameters with a range exceeding two orders of magnitude, sampling occurred on a log-scale. All other parameters remain fixed at their baseline values listed in.

The baseline and distribution of the viral decay rate was derived from by uniformly varying temperature (0–30 °C), humidity (0–100%), and UV irradiance (0–1.9 W/m^2^) as inputs to the predictive model proposed by Dabisch [43]. The probability of infection ($P_{infect}$) was chosen as the output of interest; that is, eFAST is used here to understand how changes in the selected parameters influence predictions of $P_{infect}$. Results were obtained using coughing and breathing as the emission mechanism, with the latter scenario serving as validation.

Each parameter was selected to be representative of SARS-CoV-2 and a COVID-19 infection. The literature review resulted in parameter selection; the reference used for each parameter is given in the tables. Parameters that define the scenario were not selected for variation. Room size, exposure duration, and other parameters from Table 1 are scenario-dependent parameters, and not parameters that are specific to SARS-CoV-2. The parameters in Table 2 are specific to SARS-CoV-2 particle generation, transport, and infectivity, and for the purpose of the sensitivity analysis are ideal parameters for use within a sensitivity analysis. For the parameters included in the sensitivity analysis, we attempted to decouple the infectious individual from the susceptible individual. For example, we selected different baseline minute ventilations for the infectious and susceptible individuals; however, we used the same distribution of minute ventilations for both the infectious and susceptible individuals.

eFAST uses total and first-order sensitivity indices to measure the sensitivity of output to the parameters of interest. First-order sensitivity indices capture the contribution of the individual parameter to the overall output variability, whereas the total indices measure the individual contribution of the parameter as well as that resulting from its interaction with other model parameters. Both indices lie in the interval [0, 1], with values closer to one indicating a greater influence on variation in the predicted output. When nonlinearities are prevalent in a model, as is the case here, the total sensitivity index is the most appropriate measure to consider when examining sensitivity of model parameters [64]. Consequently, results presented herein are in terms of the total sensitivity index. The first-order results are provided in the Appendices. In addition to computing sensitivity indices, eFAST allows for significance testing to determine whether the influence of a parameter on a model output can be considered statistically notable. A detailed treatment of the method itself and accompanying statistical significance testing can be found in Marino, 2008 [58].

Here, eFAST is performed using 993 simulations per parameter, with each parameter resampled 15 times, totaling 163,845 simulations. Statistical significance was established using a two-sample *t*-test applied to the parameter resampling with a significance level of α=0.01. To reduce the likelihood of incorrectly identifying a non-influential model parameter as significant, a Bonferroni correction factor was applied [65]. The sensitivity analysis was executed in MATLAB, adapting to MATLAB implementation of eFAST for biological systems consisting of ordinary differential equations by Marino, et al. [58] to suit our computational framework.

## 3. Results

Using the scenario specified in Section 2, with parameters specified in Table 1 and Table 2, numerical simulations were first performed to illustrate the dynamics exhibited in each of the submodels and the full framework for SARS-CoV-2/COVID-19. Additionally, we sought to identify the parameters that are drivers of transmissibility with a particular interest in parameters where exact values remain unknown, allowing for uncertainty to be introduced into the final predictions. Such influential parameters can be discovered by performing a sensitivity analysis, which aims to quantify how fluctuations in parameters impact a model’s behavior [66].

### 3.1. Numerical Simulations

**Figure 2:**
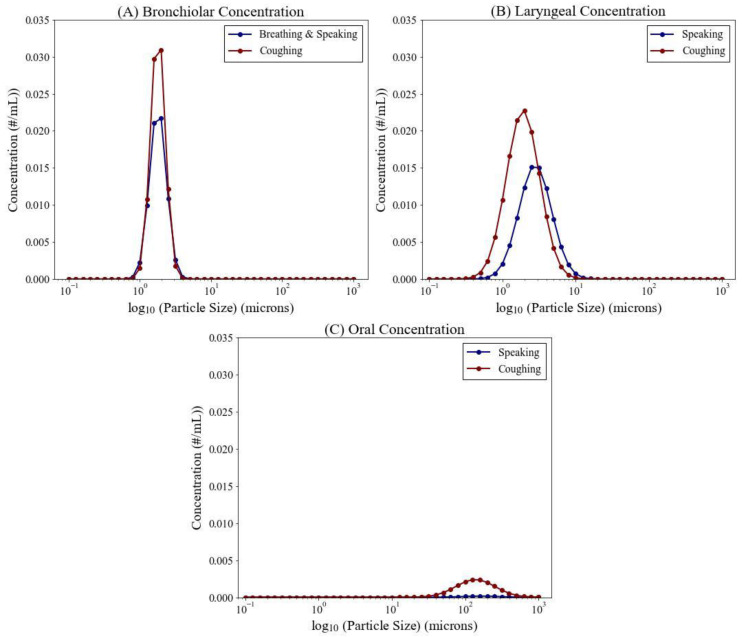
Particle concentrations versus particle size for different emission mechanisms (breathing, speaking, and coughing). Subplots (**A**–**C**) describe the concentrations emitted from the bronchiolar, laryngeal, and oral regions, respectively.

*Particle Generation.* Figure 2 shows plots of the particle concentrations emitted from each region of the respiratory tract via breathing, speaking, and coughing for varying particle sizes, which is input to the particle generation submodel. Particle concentrations from the bronchiolar (Figure 2A) and laryngeal (Figure 2B) sites encompass both small (sub-micron) and large (5 micron) particle sizes.

**Figure 3:**
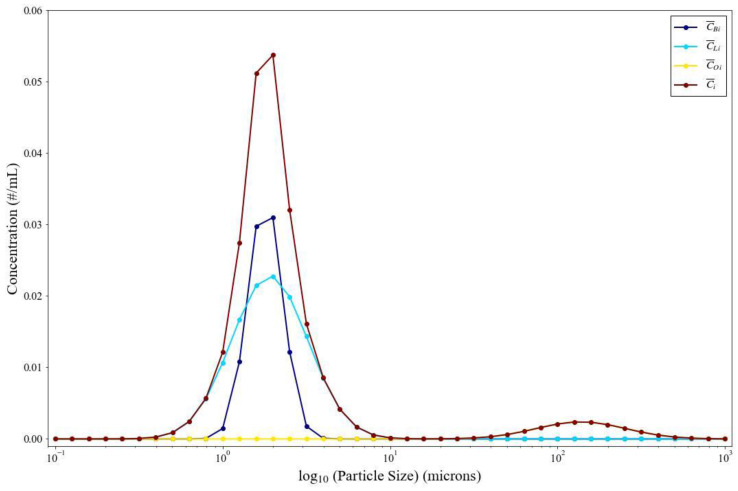
Contribution of emission route to the total particle concentration for coughing.

**Figure 4:**
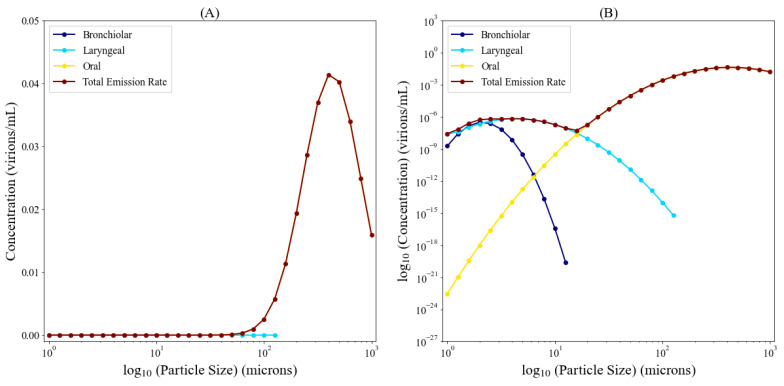
Contribution of emission route to the constant viral emission rate for coughing in linear-log space (**A**) and log-log space (**B**).

Figure 3 is calculated from data from Figure 2 and demonstrates the particle concentration contributions from the bronchiolar (${\bar{C}}_{Bi},$ dark blue), laryngeal (${\bar{C}}_{Li},$ light blue), and oral (${\bar{C}}_{Oi},$ yellow) regions during coughing as well as the total particle concentration (${\bar{C}}_{i},$ brown). Most particles generated via the respiratory tract are below ten microns (µm) (from the bronchiolar and laryngeal regions) with a non-negligible concentration above 20 µm from the oral region. The larger particles from the oral region are expected to settle rapidly, however particles in the 20 µm range can remain suspended in the air at a low concentration. Figure 4 shows the viral emission rates, further extrapolated from Figure 3. Since the larger particle sizes carry orders of magnitude more virus than the lower particle sizes, we are showing this in linear-log space (A) and log-log space (B). Low viral emission rates occur predominantly for particle sizes smaller than 0.5 µm, suggesting that they are unlikely to contain an infectious virus. Meanwhile, the majority of expelled virus takes place for particle sizes over ten microns, where we see an order of magnitude increase in viral concentration of particles every 20 microns. 

**Figure 5:**
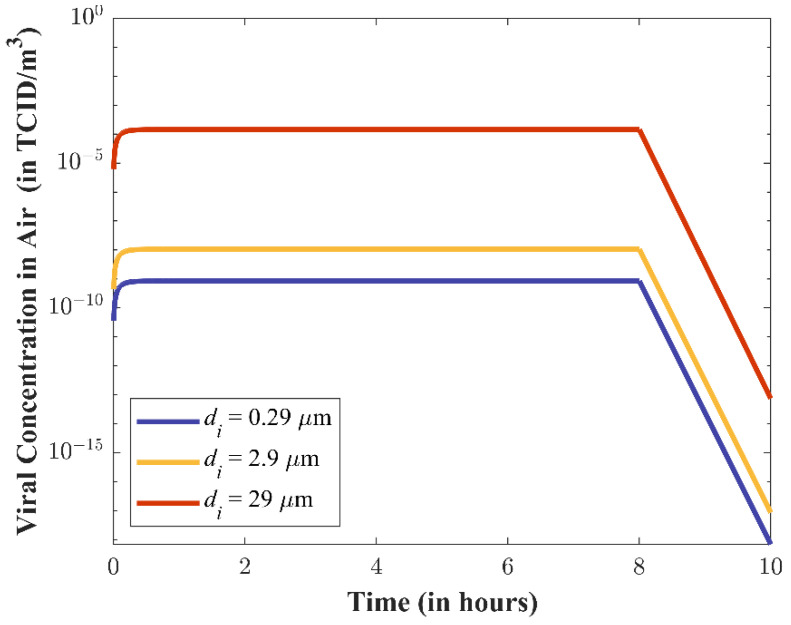
Time-series predictions for the viral concentration in the air of the indoor room ($C_{vi}\left(t\right)$) produced by the Particle Transport submodel during coughing.

*Particle Transport.* Figure 5 presents the calculated viral concentration in the air as a function of time ($C_{vi}\left(t\right)$) for different particle diameters of increasing size (0.29 µm, 2.9 µm, and 29 µm). Viral concentration increases as particle size increases, which is a byproduct of this same relationship being present in the viral emission rates (see Figure 4). The larger particles will settle faster. The viral concentration of the remaining larger particles, however, will remain several orders of magnitudes higher than the smaller particles.

**Figure 6:**
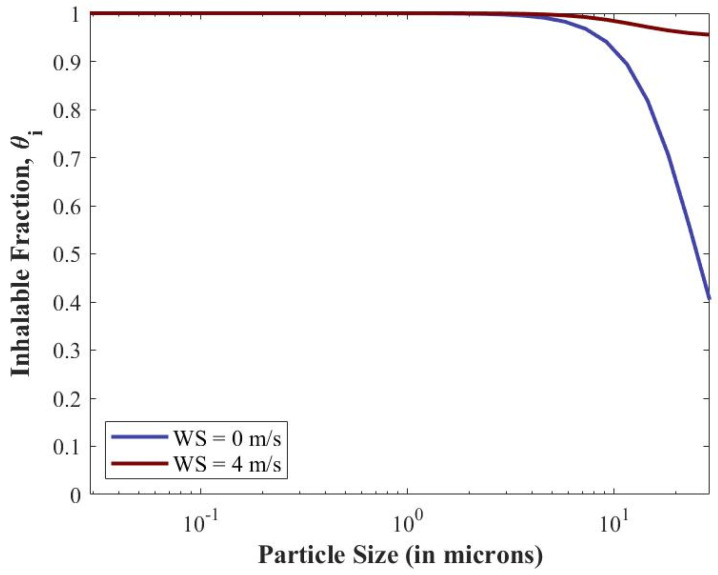
Inhalable fractions ($\theta_{i}$) for varying particle sizes ($d_{i}$ ).

*Human Response.* The inhalable fractions for different particle sizes are shown in Figure 6 and are input to the human response submodel. Since wind speed for the baseline scenario (and most indoor scenarios) is set at 0 m/s, the resulting rapid drop-off of inhalability for larger particles is expected (blue curve); however, it is worth noting that for $d_{i}=20\;\mu m$, ~65% of the particles are still inhalable. Particles with this diameter will have orders-of-magnitude higher viral concentration than smaller particles that are 100% inhalable. If the windspeed is increased, the inhalability of large particles increases as shown with the red curve. At 4 m/s, 20 μm particles have an ~95% inhalability; this will be a significant factor outside, where large particles will travel further and have a higher likelihood of being inhaled.

**Figure 7:**
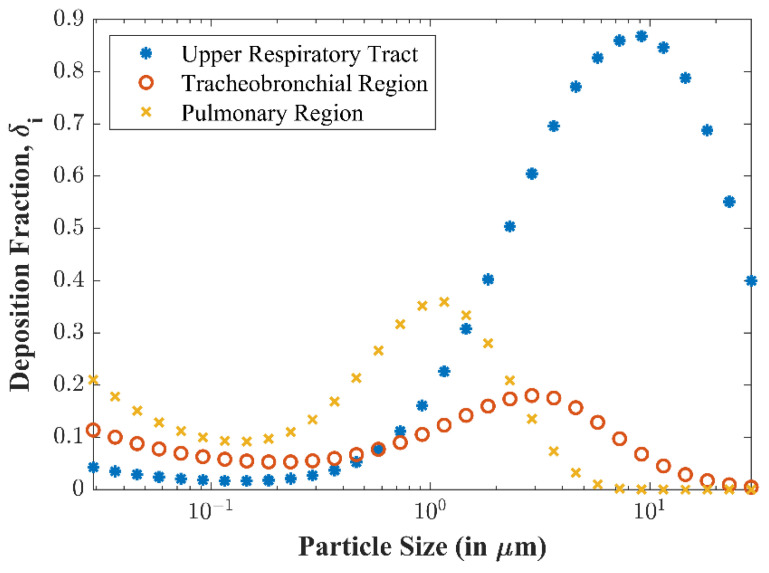
Deposition fractions ($\delta_{i}$) at different regions for varying particle diameters ($d_{i}$).

Figure 7 plots the deposition fractions (including inhalability) at the upper respiratory tract, tracheobronchial region, and pulmonary region versus particle size, calculated by the human response submodel. Observe that larger particles are unlikely to be deposited in the tracheobronchial and pulmonary airways and will instead be filtered out by the upper respiratory tract. As demonstrated above, a decrease in inhalability results in a decrease of overall deposition for larger particles.

**Figure 8:**
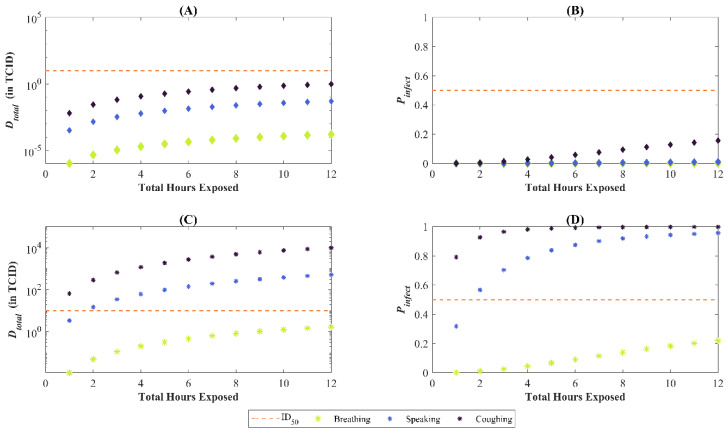
Total deposited dose ($D_{total}$) and probability of infection ($P_{infect}$) for varying continuous exposure periods. The orange dashed line in each subplot represents the ID_50_ or 50% chance infection. Subplots (**A**,**B**) correspond to the median values, whereas (**C**,**D**) to the 95th percentile. In our baseline scenario, this is only met for the 95th percentile of $D_{total}$ (or $P_{infect}$) and if coughing or speaking is the particle emission mechanism into the environment by the infectious person.

**Figure 9:**
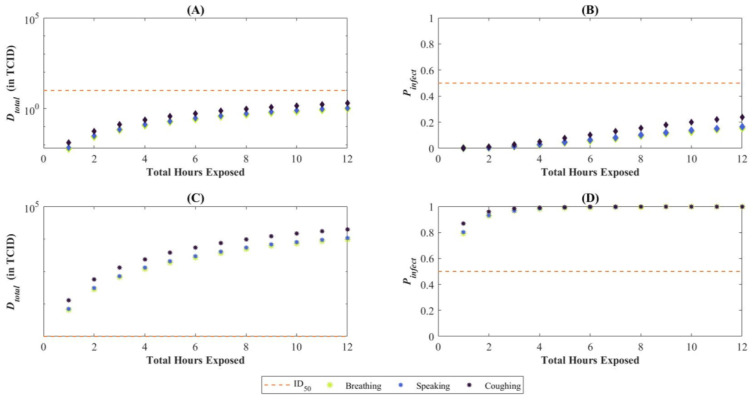
Total deposited dose (D_total_) and probability of infection (P_infect_) for varying continuous exposure periods with ventilation turned off. The orange dashed line in each subplot represents the ID50 or 50% chance infection. Subplots (**A**,**B**) correspond to the median values, whereas (**C**,**D**) to the 95th percentile.

Now, suppose the exposure period of the SARS-CoV-2 susceptible person is varied but does not exceed 12 h; that is, $t_{E2}$ is no longer fixed at six hours but instead, $t_{E2}\in \left[0,12\right]$. The susceptible person is still at a distance of greater than 2 m from the infectious person, so the only exposure continues to be via the steady-state concentration. The TCID_50_ used in this analysis is of the same order as that for symptomatic presentation of disease [17] for the alpha variant of SARS-CoV-2. The subplots (B) and (D) of Figure 8 display how the median and 95th percentile for the Probability of Infection ($P_{infect}$) change as the exposure period increases for virus emitted via coughing, speaking, and breathing. If the infectious person in the room is coughing, the probability of infection reaching approximately 50% after an hour even if the susceptible person is not directly interacting with the infectious person. After seven hours, that likelihood of infection increases to 90% for the 95th percentile. Subplots (A) and (C) of Figure 8 display analogous results for the total deposited dose, $D_{total}$. The results accurately capture how infection risk is greater under coughing as compared to breathing. Figure 9 shows the same outputs for a scenario wherein the ventilation in the room is turned off. Whereas the total cumulative dose increases in (A) and (C), we only see an appreciable increase in the Probability of Infection for the 95th percentile case, where all modes of particle emission result in a 50% probability of infection within two hours of exposure. 

### 3.2. Sensitivity Analysis

**Figure 10:**
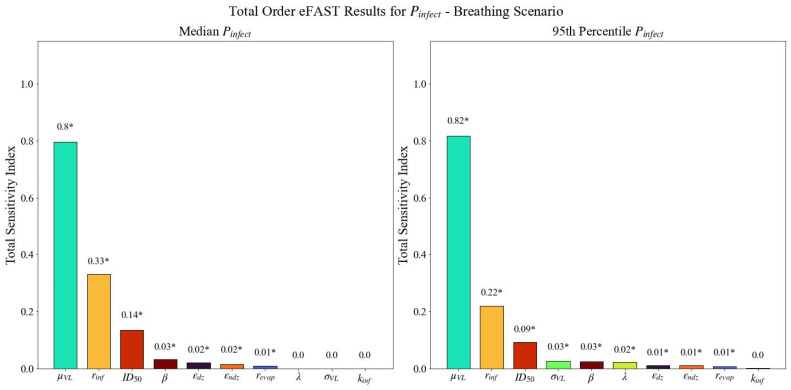
Total sensitivity indices (ordered from largest to smallest) derived using eFAST for the median $P_{infect}$ and 95th percentile of $P_{infect}\;$ with breathing as the emission mechanism. Asterisks (*) indicate statistically significant sensitivity indices (α = 0.01).

**Figure 11:**
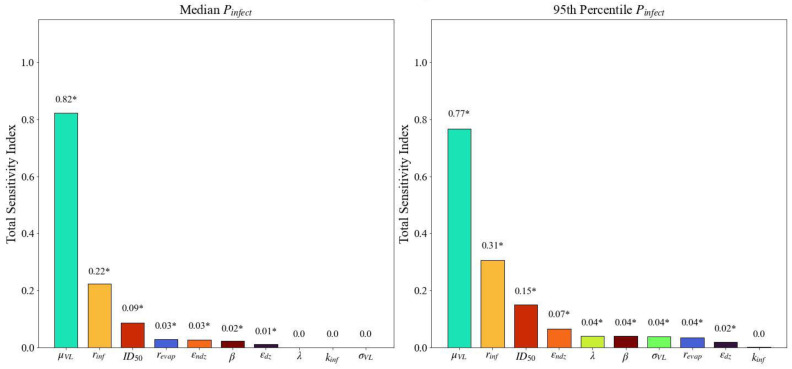
Total sensitivity indices (ordered from largest to smallest) derived using eFAST for the median $P_{infect}$ and 95th percentile of $P_{infect}$ with coughing as the emission mechanism. Asterisks (*) indicate statistically significant sensitivity indices (α=0.01).

The total sensitivity indices produced by eFAST for the median and 95th percentile of $P_{infect}$ resulting from exposure to SARS-CoV-2 contaminated particles emitted by an infectious person breathing and coughing are presented in Figure 10 and Figure 11, respectively. The parameter rankings are displayed in Table 3.

Note that there are noticeable consistencies between the two outcomes (median and 95th percentile $P_{infect}$) for the two emission methods (breathing and coughing), both in the rankings of the parameters’ sensitivity indices and in the set of parameters identified as influential. With respect to all outputs, $\mu_{VL}$, $r_{inf}$, $r_{evap}$, $ID_{50}$, $\varepsilon_{ndz}$, β, and $\varepsilon_{dz}$ were identified as statistically significant, suggesting variability in the median $P_{infect}$ is sensitive to changes in these parameters. $\sigma_{VL}$ and λ were also identified as statistically significant for the 95th percentile of $P_{infect}$. Visual inspection of Figure 11 reveals total sensitivity index for $\mu_{VL}$ (the median viral load in the infectious person) is clearly delineated from those for the other parameters for all four emission routes and outcomes. The parameter ranked as the second most influential, $r_{inf}$ (the ratio of RNA copies to live virions), is also distinct from the remaining parameters. While less extreme than that of $\mu_{VL}$, the magnitude of this difference is still readily evident. In all cases, the $ID_{50}$ (the median infectious dose) is the third most influential parameter. The consistency of these results between the two outcomes strengthens the validity of their importance.

Observe that, with respect to the 95th percentile $P_{infect}$, total sensitivity indices of the $\sigma_{VL}$ and λ were also found to be statistically significant, but not for the median; this is an expected consequence of the model design, as these two parameters are involved only in the calculation of the 95th percentile $P_{infect}$, and not in that of median $P_{infect}$, further reinforcing the credibility of the results.

The first-order indices for coughing identified similar parameters as statistically significant as the total sensitivity indices with one exception (results not shown; see Appendix B). The probit slope β was not identified as significant for the median $P_{infect}$ for the coughing scenario. It is common that there are slight differences between parameters identified as statistically significant by this index when compared to the total index. The first-order indices for breathing identified fewer statistically significant parameters than total sensitivity indices for both the median and 95th percentile $P_{infect}$. The first-order sensitivity analysis did not identify $r_{evap}$ as statistically significant for the median $P_{infect}$. β was not identified as statistically significant for the 95th percentile $P_{infect}$. We note that $\mu_{VL}$, $r_{inf}$, and $ID_{50}$ remain ranked as first, second, and third most influential parameters, respectively in both the first-order and total sensitivity index for breathing and coughing emissions.

## 4. Discussion

The results presented in Section 3 provide insight into the inputs and outputs of an end-to-end, particle size-inclusive disease transmission model. We will discuss the results of the individual submodels before discussing the overall parameter sensitivities.

### 4.1. Numerical Simulations

*Particle Generation*. The particle generation results begin to show one of the core reasons to include a range of particle sizes in analysis: large particles are generated, and they carry orders of magnitude more virus than smaller particles. The viral load is driven by several factors that can be included in the model such as the viral load in the individual (which would be a function of the individual and their time since exposure); however, an underlying assumption could be made that the ratio of virions in small to large particles will be consistent regardless of those factors. Considering risk to a susceptible person, inhaling large particles would result in an inhaled dose of infectious agent that would be several orders of magnitude higher than that of the smaller, more abundant particles, resulting in a higher likelihood of infection. Large particles are often discounted from analysis as they tend to settle quickly or are not as inhalable as smaller particles. While both arguments are true, the expected viral load in these large particles greatly outweighs increased settling or decreased inhalability; this phenomenon is shown in the Particle Transport submodel. One consideration that we did not include in our analysis was the dependence of initial jet flow dynamics and turbulence on the respiratory dynamics driving the emission events (e.g., normal breathing or coughing and sneezing [67]); these events can cause additional lifting of larger particles, eventually resulting in settling further away from the infected individual.

*Particle Transport*. The particle transport submodel shows how the presented viral concentration over time is largely driven by particle size. Using FaTIMA, simulation results show that larger particles (29 µm) remain suspended in the air long enough to present approximately four orders-of-magnitude higher viral concentration than smaller particles (0.29 and 2.9 µm) despite contributing an order of magnitude fewer total particles to the presented concentration; this alone inherently shows the risk of being collocated in a room with an individual who is coughing, even if standing away from the infectious individual. Modeling suites such as FaTIMA have been used to estimate indoor concentrations; however, they are not designed to handle complex materials (i.e., multiple particle sizes, wet/dehydrating particles) that include multiple sources, particle size distributions, and particle composition, nor do they account for evaporation of the volatile water content in respiratory particles. To account for this, we used post-evaporation particle sizes in our implementation of FaTIMA. Another factor not considered is proximity to the infectious individual. In this scenario we considered a susceptible individual standing far enough away from an individual that only the steady-state concentration in the room would contribute to the presented concentration. One can surmise that if an individual is closer to the emitter, the concentration of larger particles will be greater as they have not settled, and the likelihood of infection would increase at shorter exposure times.

*Human Response*. The particle transport submodel demonstrated the particle sizes and viral concentrations to which a susceptible individual could be exposed. The human response submodel showed the inhalability of those particles and where they deposit before considering infectivity. The results show that large particles (greater than 10 microns) are still inhalable, with over 65% being inhaled. Additionally, large particles are more likely to deposit once inhaled, whereas smaller particles can be exhaled. The results in Figure 7 show this phenomenon; smaller particles with 100% inhalability only have 60% or less deposition in the respiratory tract. Conversely, larger particles that have a 60% or greater inhalability fraction have a 100% deposition in the respiratory tract.

The results also show which region particles deposit in as a function of size, with larger particles depositing in the upper respiratory tract and smaller particles deposited primarily in the pulmonary and tracheobronchial region; this is an important phenomenon, as evidence of decreased infectivity for biological agents deposited in the head has been shown for several pathogens and toxins; this has not yet been shown to be the case experimentally for COVID-19 but is an area of additional uncertainty that could be included in the model.

The model also did not account for disease severity. Dabisch [17] showed a difference in the ID_50_ for seroconversion vs. illness which is one aspect of severity that can account for asymptomatic individuals but does not account for differences in severity for symptomatic individuals. Typically, severity models are developed from observation to the distribution of severities across demographic cohorts. A greater understanding of host immune response and extrapolation between species could eventually result in predictive models of disease severity at the early stages of an epidemic.

### 4.2. Sensitivity Analysis

There are mathematical and physiological explanations for the results obtained by eFAST. Recall from Section 3 that the three most important model parameters, as determined by eFAST, are the median viral load ($\mu_{VL}$), infectivity ratio ($r_{inf}$), and median infectious dose ($ID_{50}$). It is worth noting that the median viral load is a parameter that will vary widely from one individual to the next and that the infectivity ratio is a value that has not been well characterized for SARS-CoV-2. The $ID_{50}$ is a parameter that has been characterized in humans. Physiologically, the magnitude of viral particles generated across individuals are distributed lognormally, with super spreaders shown to generate orders of magnitude more virus than a mean spreader. The introduction of $\mu_{VL}$ at the beginning of the end-to-end framework leads to its variability and influence being recognized within each submodel, from particle generation through human effects. By assuming a size-independent concentration of virions in the emitted particles, larger particles would be laden with significantly more live virions whereas smaller particles would still be limited by the size of the virion compared to the volume of the particle. The authors note that a recent study found that particles over 4 microns generated in six hospital rooms contained RNA copies but not evidence of replicating virus in these droplets [68]. The author hypothesized that particles generated in the respiratory tract are more likely to be culturable. The model as presented could be expanded in the future to include a particle-size dependent concentration of RNA copies or ratio of RNA copies to live viruses. The significance of the infectivity ratio ($r_{inf}$) is likely a result of its use being to estimate the number of live virions as a function of RNA copies, a value that directly influences the number of inhaled virions by a susceptible individual. $\mu_{VL}$ and $r_{inf}$ are parameters with an understood wide range of values and are positioned at the beginning of the end-to-end model; their influence on the output is expected. The $ID_{50}$ is used at the end of the calculation to estimate probability of infection for a deposited dose; since probability of infection is the output of the model, the $ID_{50}$ being identified as an influential parameter makes logical sense. Understanding the influence of these parameters early in the pandemic could have been a driving force to prioritizing research.

Observations can be made when comparing across emission mechanisms. The ranked order of influential parameters is consistent for the top three parameters ($\mu_{VL}$, $r_{inf}$, and $ID_{50}$) when comparing breathing to coughing. The fourth most influential parameter for breathing is the probit slope, whereas the fourth most influential parameter for coughing is a measure of particle size as it pertains to evaporation. The intuitive difference is in the effect of evaporation on small particles that result from breathing compared to large particles that result from coughing. For the larger particles, the evaporation dictates settling velocity and presented concentration. For the smaller particles, evaporation does decrease the size of the particle and its settling velocity, but the settling velocity is already very small and has an insignificant effect on the airborne concentration.

It is also worth noting the probit slope (β) was consistently less influential in the first-order sensitivity indices than in the total sensitivity indices. For the first-order sensitivity indices of the median $P_{infect}$ and the 95th percentile $P_{infect}$, β was not identified as a statistically significant parameter. The influence of β can be surmised when Figure 8, particularly noting the deposited TCID_50_ across the different scenarios using the baseline values. Table 4 shows a summary of the approximate TCID_50_ alongside the sensitivity ranking and the statistical significance of β. The two scenarios where the baseline values result in a deposited TCID_50_ of approximately 1 both resulted in β not being identified as significant. A deposited dose of 1 TCID_50_ is close to the baseline ID_50_ of 10 TCID_50_, and changes to the probit slope will not have a great effect on the outcome, especially considering the probit slope distribution of 0.25 to 1.5 as specified in Table 2. Conversely, the two scenarios where β was identified as significant had deposited doses of 1 × 10^4^ and 1 × 10^−4^ TCID_50_. Deposited doses this far from an ID_50_ of 10 TCID_50_ will be more sensitive to the probit slope.

It is notable that eFAST identifies $k_{inf}$, the viral decay rate, as a not significantly sensitive parameter across all scenarios; this could be due to our scenario: the emission of particles by a single individual for a period of time within an enclosed, well-ventilated space. Virus-laden particles were constantly being added to the environment and the viral decay rate was not rapid enough to depreciate the viral concentration considerably compared to removal by settling or ventilation. The significance of this parameter could obviously change in outdoor environments, where UV intensity is a significant driver of viral decay.

The influential parameters identified herein are deemed as such by eFAST because—by design—fluctuations in their value disseminate to a significant amount of variation in model output; ergo, uncertainty in their values will propagate to uncertainty in model output. Gaining a better understanding of these influential parameters and obtaining accurate estimates of their values will help to inform and prioritize future research, with the goal of reducing uncertainty in subsequent risk assessment and mitigation techniques.

## 5. Conclusions

In this paper, we present a robust modeling framework of person-to-person disease spread for respiratory transmitted biological pathogens that considers disease transmissibility using three submodels: (1) particle generation; (2) transport and dispersion; and (3) human response (i.e., infectivity and severity of symptoms/clinical presentation of infected individuals). Although developed and validated using data generated and collected from SARS-CoV-2 and COVID-19 studies, the framework is designed to work for a range of pathogens and particle sizes and estimates the generation rate, environmental fate, deposited dose, and infection/severity. Thus, it provides a generalizable, threat-agnostic, end-to-end analytic approach that can be applied to emerging communicable infectious respiratory pathogens of varying particle sizes and can be transitioned to individual and population health models accounting for varying levels of disease severity. A global sensitivity analysis performed on the model framework identified critical parameters associated with respiratory disease throughout the transmission process; this knowledge can be used to advance and prioritize research efforts by highlighting key parameters of the transmission cycle needing rigorous quantification. Further, the outputs of the end-to-end analytic framework can also be applied to inform operational decision-making by identifying which stages of the disease transmission life cycle should be targeted for optimal risk mitigation and effective risk management; this framework ultimately adds value for risk analysis considering an emerging contagious disease and is complemented by a parameter sensitivity analysis to highlight critical parameters, categorize uncertainty, and prioritize gaps.

## Figures and Tables

**Table 1:** Parameters and values used for baseline scenario.

Symbol	Description	Units	Baseline	Refs.
$\rho_{d}$	Particle density	$g/cm^{3}$	1	Assumed
*Particle Generation*				
$t_{G1}$	Time at start of particle generation	h	0	Assumed
$t_{G1}$	Time at end of particle generation	h	8	Assumed
$CMD_{B}$	Count median diameter of particles generated in the bronchiolar region	μm	1.6	[36]
$GSD_{B}$	Geometric standard deviation of particles generated in the bronchiolar region	μm	$\{\begin{array}{c} \begin{array}{cc} 1.30, & if\;breathing \end{array}\\ \begin{array}{cc} 1.30, & if\;speaking \end{array}\\ \begin{array}{cc} 1.25, & if\;coughing \end{array} \end{array}$	[36]
$\eta_{B}$	Number concentration of particles generated in the bronchiolar region	#/mL	$\{\begin{array}{c} \begin{array}{cc} 0.069, & if\;breathing \end{array}\\ \begin{array}{cc} 0.069, & if\;speaking \end{array}\\ \begin{array}{cc} 0.087, & if\;coughing \end{array} \end{array}$	[36]
$CMD_{L}$	Count median diameter of particles generated in the laryngeal region	μm	$\{\begin{array}{c} \begin{array}{cc} NA, & if\;breathing \end{array}\\ \begin{array}{cc} 2.5, & if\;speaking \end{array}\\ \begin{array}{cc} 1.7, & if\;coughing \end{array} \end{array}$	[36]
$GSD_{L}$	Geometric standard deviation of particles generated in the laryngeal region	μm	$\{\begin{array}{c} \begin{array}{cc} NA, & if\;breathing \end{array}\\ \begin{array}{cc} 1.66, & if\;speaking \end{array}\\ \begin{array}{cc} 1.68, & if\;coughing \end{array} \end{array}$	[36]
$\eta_{L}$	Number concentration of particles generated in the laryngeal region	#/mL	$\{\begin{array}{c} \begin{array}{cc} NA, & if\;breathing \end{array}\\ \begin{array}{cc} 0.086, & if\;speaking \end{array}\\ \begin{array}{cc} 0.130, & if\;coughing \end{array} \end{array}$	[36]
$CMD_{O}$	Count median diameter of particles generated in the oral region	μm	$\{\begin{array}{c} \begin{array}{cc} NA, & if\;breathing \end{array}\\ \begin{array}{cc} 145, & if\;speaking \end{array}\\ \begin{array}{cc} 123, & if\;coughing \end{array} \end{array}$	[36]
$GSD_{O}$	Geometric standard deviation of particles generated in the oral region	μm	$\{\begin{array}{c} \begin{array}{cc} NA, & if\;breathing \end{array}\\ \begin{array}{cc} 1.80, & if\;speaking \end{array}\\ \begin{array}{cc} 1.84, & if\;coughing \end{array} \end{array}$	[36]
$\eta_{O}$	Number concentration of particles generated in the oral region	#/mL	$\{\begin{array}{c} \begin{array}{cc} NA, & if\;breathing \end{array}\\ \begin{array}{cc} 0.001, & if\;speaking \end{array}\\ \begin{array}{cc} 0.016, & if\;coughing \end{array} \end{array}$	[36]
*Particle Transport*				
κ	Dynamic viscosity of air	cm⋅s	0.000181	-
g	Gravitational constant	$cm/s^{2}$	981	-
V	Volume of the room	$m^{3}$	100	Assumed
$A_{floor}$	Surface area of the room floor	$m^{2}$	25	Assumed
$Q_{r}$	Return ventilation volumetric airflow rate	$m^{3}$/s	0.0278	Assumed
*Human Response*				
$t_{E1}$	Time at beginning of exposure	h	0	Assumed
$t_{E2}$	Time at end of exposure	h	6	Assumed
*WS*	Wind speed	m/s	0	Assumed

**Table 2:** Parameters with distributions for sensitivity analysis.

Symbol	Description	Units	Baseline Value	Distribution	Refs.
*Particle Generation*					
$\varepsilon_{dz}$	Respiratory minute ventilation of the infectious person	L/min	15	Uniform (5, 75)	Assumed
*Particle Transport*					
$r_{evap}$	Ratio of evaporated particle diameter to initial particle diameter	-	0.29	Uniform (0, 1)	[48]
$k_{inf}$	Viral decay rate	$min^{-1}$	0.1577	Normal (0.1614, 0.0863)	Derived from [43]
$\mu_{VL}$	Median viral load	$log_{10}\;RNA\;copies/mL$	7.19	Uniform (3, 13)	Derived from [63]
$\sigma_{VL}$	Standard deviation of the viral load for the infectious individual	$log_{10}\;RNA\;copies/mL$	1.35	*Normal (1.58, 0.22)*	Derived from Ref. [63]
λ	Super spreader emission factor	-	20	*Uniform (0, 40)*	[52]
$r_{inf}$	Infectivity ratio	RNA copies/TCID	10^2^	$Uniform(10^{1}$ $,10^{6})$	[23,24]
*Human Response*					
$\varepsilon_{ndz}$	Respiratory minute ventilation of the SARS-CoV-2 susceptible person	L/min	10	Uniform (5,75)	Assumed
$ID_{50}$	Median infectious dose	$TCID_{50}$	10	$Uniform\;\left(10^{-1},10^{4}\right)$	[17]
β	Base-10 probit slope for the probability of infection	$log_{10}$	1	Uniform (0.25, 1.5)	[17]

**Table 3:** Ranking of the total sensitivity indices for the median and 95th percentile $P_{infect}$ with breathing and coughing as the emission mechanisms. Yellow shaded cells indicate parameters that were determined to be statistically significant.

Parameter	Parameter Description	Total Sensitivity Index Ranking			
		$\mathrm{Median}\;P_{infect}$		$95\mathrm{th}\;\mathrm{Percentile}\;P_{infect}$	
		Breathing	Coughing	Breathing	Coughing
$\varepsilon_{dz}$	Respiratory minute ventilation of the infectious person	5	7	7	9
$r_{evap}$	Ratio of evaporated particle size to initial particle size	7	4	9	8
$k_{inf}$	Viral decay rate	10	9	10	10
$\mu_{VL}$	Median viral load	1	1	1	1
$\sigma_{VL}$	Standard deviation of the viral load for the infectious individual	9	10	4	7
λ	Super spreader emission factor	8	8	6	5
$r_{inf}$	Infectivity ratio	2	2	2	2
$\varepsilon_{ndz}$	Respiratory minute ventilation of the SARS-CoV-2 susceptible person	6	5	8	4
$ID_{50}$	Median infectious dose	3	3	3	3
β	Base-10 probit slope for the probability of infection	4	6	5	6

**Table 4:** Deposited dose and sensitivity index ranking/significance for β across multiple scenarios.

Scenario	~Deposited Dose (TCID_50_)	Ranking (Statistically Significant)
Coughing/Median $P_{infect}$	1	7 (Insignificant)
Coughing/95th percentile $P_{infect}$	1 × 10^4^	8 (Significant)
Breathing/Median $P_{infect}$	1 × 10^−4^	5 (Significant)
Breathing/95th percentile $P_{infect}$	1	9 (Insignificant)

**Figure A1:**
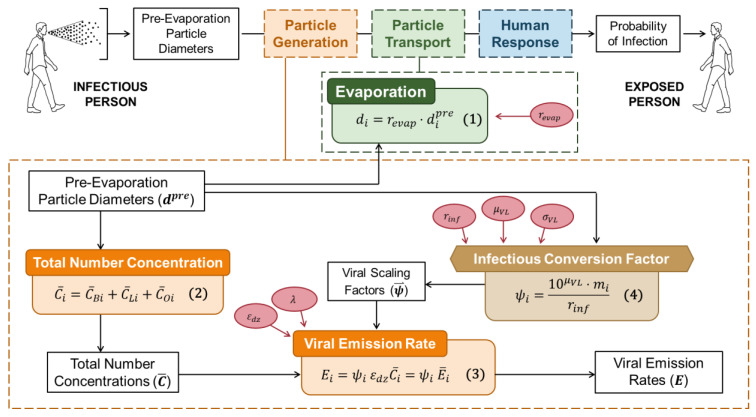
Visualization of the particle generation submodel workflow. Due to the rapid time scale at which it occurs (i.e., within the first 50 s), near-field dynamics of the Particle Transport submodel (Evaporation box in green) is displayed alongside the Particle Generation submodel. Red circles indicate parameters selected for sensitivity analysis (see Section 2). All notation definitions are provided in Table 1.

**Figure A2:**
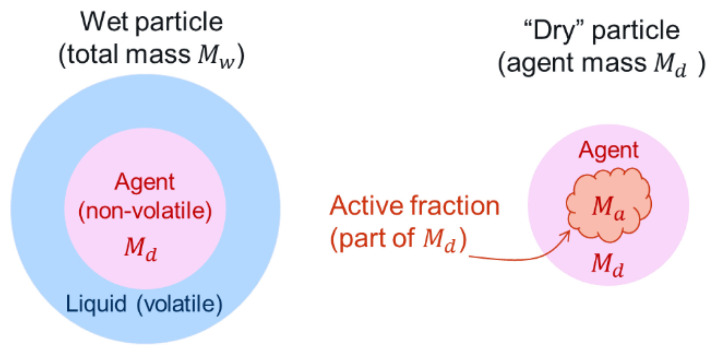
Simplified schematic of a particle.

**Figure A3:**
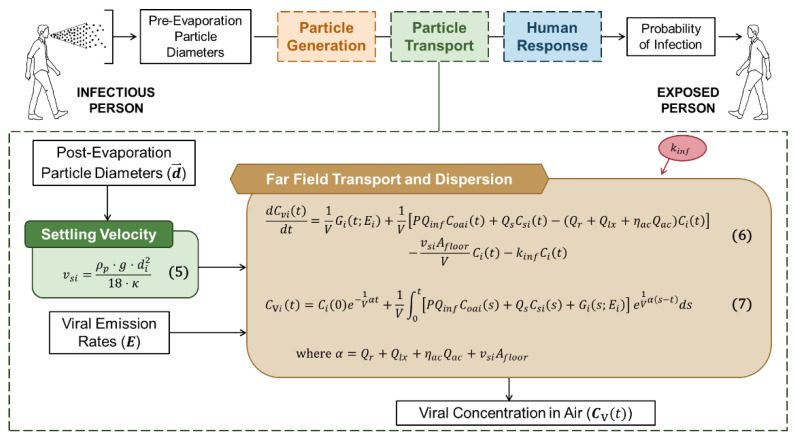
Visualization of the aerosol transport submodel workflow. Equations (6) and (7) are equivalent as the latter is the analytical solution of the former. When executing the workflow, either can be utilized, however, Equation (7) is preferred as the complexity of the numerical integration is reduced, in turn minimizing both the numerical errors introduced and the computational time required. Red circles indicate parameters selected for sensitivity analysis (see Table 2).

**Figure A4:**
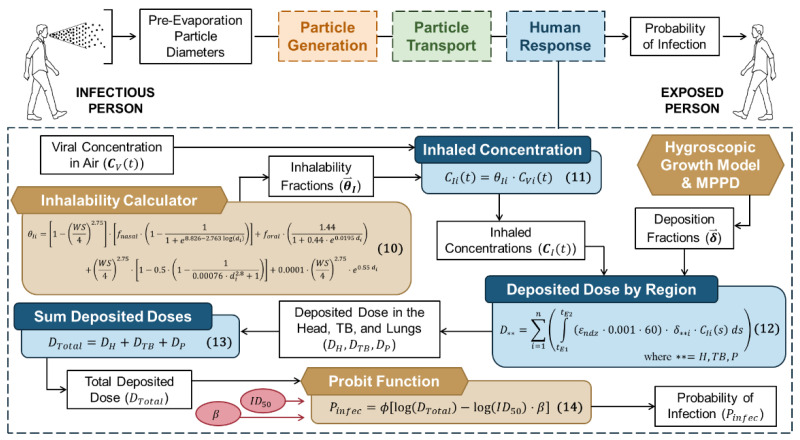
Visualization of the human response submodel workflow. Red circles indicate parameters selected for sensitivity analysis (see Section 2). All notation definitions are provided in Table 1.

**Figure A5:**
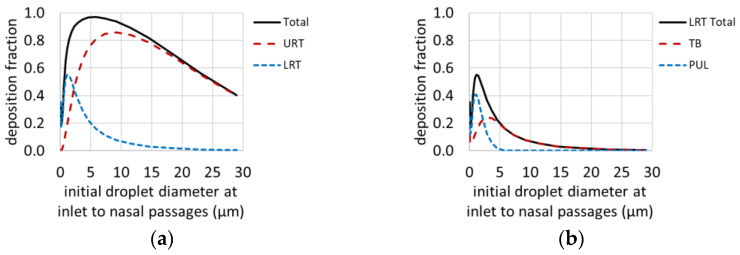
Plots of example look-up table data of particle deposition fraction versus initial diameter of a particle at the inlet to the nasal passages of a susceptible person including deposition in (**a**) the URT (red), LRT (blue), and total URT and LRT (black), and (**b**) the tracheobronchial region (TB) (red), pulmonary region (PUL) (blue), and total TB and PUL, or LRT (black). At the inlet to the nasal passages, initial properties are a particle composition of 74% well-mixed nonvolatile components (NaCl) and 26% water by mass, air and particle temperatures of 20 °C, and relative humidity of 50%.

**Figure A6:**
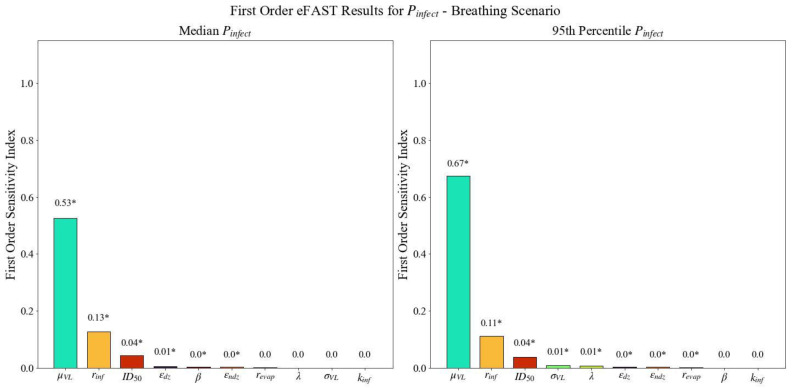
First-order sensitivity indices (ordered from largest to smallest) derived using eFAST for the median $P_{infect}$ and 95th percentile of $P_{infect}$ with breathing as the emission mechanism. Asterisks (*) indicate statistically significant sensitivity indices (α = 0.01).

**Figure A7:**
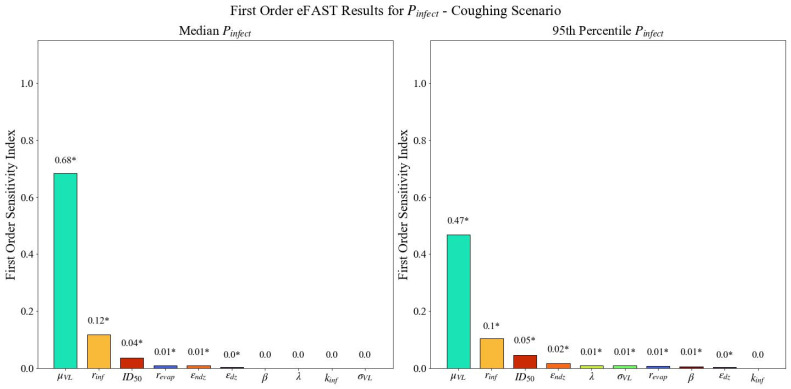
First-order sensitivity indices (ordered from largest to smallest) derived using eFAST for the median $P_{infect}$ and 95th percentile of $P_{infect}$ with coughing as the emission mechanism. Asterisks (*) indicate statistically significant sensitivity indices (α=0.01).

## Data Availability

Python implementation of model is made available as supplementary material to this paper.

## Supplementary Materials

The following supporting information can be downloaded at: https://www.mdpi.com/article/10.3390/v14071567/s1.

## Appendix A. Detailed Model Discussion

In this appendix, we provide greater detail and the accompanying equations for the proposed end-to-end modeling framework. Following the format of the main body, we will divide this section into Particle Generation, Particle Transport, and Human Response.

### Appendix A.1. Particle Generation

The particle generation submodel characterizes the respiratory particles released into the air by an infectious individual via different emission mechanisms and the subsequent evaporation of most of the volatile liquid (water) content from the particles. Figure A1 summarizes the workflow of this submodel. The first step in developing a model of respiratory particle emission is to physically characterize the particles. Respiratory particles are heterogeneous and vary in number, size, chemical composition, and viral content depending on the generation mechanism, the stage of infection, as well as inter- and intra-subject variability [32,36,69,70]. Accounting for this heterogeneity is an important step in quantifying the potential uncertainty in particle emission from an infected individual. The physical characterization of emitted respiratory particles will allow for improved modeling of the fate of respiratory particles in the environment and inform estimates of inhalation, deposition, and infectivity within the respiratory tract of susceptible populations.

A simplified schematic of a respiratory particle as it is emitted from an infectious person is shown in Figure A2. The particle can be described as a combination of volatile liquid (such as water), non-volatile and non-soluble materials (an actively infectious component and other material, such as proteins), and non-volatile, soluble materials that are suspended in the liquid portion (such as salts). As the particle is transported, the volatile material evaporates, leaving the non-volatile and soluble portions of the particle.

A saliva particle, for example, is composed of approximately 3% well-mixed nonvolatile components and 97% water by mass [71]. The nonvolatile components are varied, as shown in Table A1. Most of a saliva particle is made up of salts (NaCl and KCl) and mucin; these components will affect the dehydration and rehydration of particles and, for a high-fidelity model, should be tracked. For dehydration, we used data from [48] and consultation with Walker to determine post-evaporation diameters from pre-evaporation diameters. We settled on a post-evaporation diameter of 29% of the original pre-evaporation saliva particle size.

**Table A1:** Components of a saliva particle. The first two columns were calculated in [48] and the other columns were calculated.

Component	Concentration (Mass per Liter of Fluid) (g/L)	Density of Component (g/L)	Volume of Component/Dry Particle Volume	Weighted Density in Dry Particle (g/L)
MgCl_2_	0.04	2320	4.52 × 10^−3^	10
CaCl_2_.H_2_0	0.013	2240	1.52 × 10^−3^	3
NaHCO_3_	0.42	2200	5.01 × 10^−2^	110
KH_2_PO_4_	0.21	2340	2.35 × 10^−2^	55
K_2_HPO_4_	0.43	2450	4.59 × 10^−2^	112
NH_4_Cl	0.11	1530	1.89 × 10^−2^	29
KSCN	0.19	1900	2.62 × 10^−2^	50
(NH_2_)_2_CO (urea)	0.12	1340	2.35 × 10^−2^	31
NaCl	0.88	2165	1.07 × 10^−1^	231
KCl	1.04	1984	1.37 × 10^−1^	273
Mucin	3	1400	5.62 × 10^−1^	787
DMEM	1 mL per liter of fluid	-	-	14
Alpha-amylase	-	-	-	-
Deionized water	979 mL per liter of fluid	-	0	0
***Sum***	**-**	**-**	**-**	***1706***

Respiratory particles are primarily generated in three locations within the human respiratory tract. Studies have characterized primary emission locations and mechanisms [36]. First, respiratory particles are generated during normal breathing and these particles are generally in the range of 1–2 µm in diameter [72]. Second, respiratory particles may also be generated in the larynx during activities such as speaking or coughing; these particles are also in the range of 1–2 µm in diameter when expelled from the respiratory tract. The final primary location of particle generation is the oral cavity; this generation mechanism is primarily associated with coughing and to a lesser extent with speaking, and these particles are much larger in size (approximately 100 µm in diameter) when measured at emission [36]. Particles generated by these three locations during speaking, coughing, and normal breathing are quantified in terms of the count median diameter (CMD), geometric standard deviation (GSD), and number concentration (η) [36]. Values for these three parameters for each respiratory activity are provided in Table A2.

**Table A2:** Size distribution of particles emitted via breathing, speaking, and coughing.

	Breathing			Speaking			Coughing		
Location	CMD (μm)	GSD	Concentration (#/cm^3^)	CMD (μm)	GSD	Concentration (#/cm^3^)	CMD (μm)	GSD	Concentration (#/cm^3^)
Bronchiolar	1.6	1.30	0.069	1.6	1.30	0.069	1.6	1.25	0.087
Laryngeal	N/A	N/A	N/A	2.5	1.66	0.086	1.7	1.68	0.130
Oral	N/A	N/A	N/A	145	1.80	0.001	123	1.84	0.016

For our implementation of the model, emission rate varies with particle size and is determined by assuming a lognormal particle size distribution combining the contributions from each source for particle size bins ranging from 0.1 µm in diameter to 100 μm in diameter. A lower and upper size limit should be selected for virus-containing particles. For example, the lower size limit for virus-containing respiratory particles of 0.1 µm can be chosen based on the 100 nm estimated size of SARS-CoV-2 and the estimated initial water content of the respiratory particles being 90% [38]. The upper size limit for virus-containing respiratory particles of 100 µm was chosen based on an estimated settling time of less than 20 s for particles larger than 100 µm in diameter. Therefore, the submodel assumes particles larger than 100 µm in diameter will deposit to surfaces quickly and are not a long-term airborne exposure risk in most situations.

For a given pre-evaporation particle diameter $d_{i}$, the total particle emission rate, ${\bar{E}}_{i}$, depends on the minute ventilation of the infected subject ($\varepsilon_{dz}$), the source of the particles (bronchiolar, laryngeal, or oral), and the emission mechanism (breathing, speaking, or coughing) within the respiratory tract. More specifically,

$${\bar{E}}_{i}=\varepsilon_{dz}\left({\bar{C}}_{Bi}+{\bar{C}}_{Li}+{\bar{C}}_{Oi}\right)$$

where ${\bar{C}}_{Bi}$, ${\bar{C}}_{Li}$, and ${\bar{C}}_{Oi}$ denote the particle number concentration emitted from the bronchiolar, laryngeal, and oral sites, respectively, as determined by the corresponding values of CMD, GSD, and η. Note that the minute ventilation $\varepsilon_{dz}$ is the product of the tidal volume and breathing frequency both of the infectious subject. The above equation for ${\bar{E}}_{i}$ is equivent to that given by Equations (2) and (3) in Figure A1.

Polymerase chain reaction (PCR) tests measure the number of RNA copies per volume of liquid sample collected; however, these viral RNA counts are not an indicator of infectiousness as they can include non-infectious viral genetic material that has been neutralized by the immune system and is cleared by the respiratory tract [34,73]. For this model framework we are interested in the infectious viral content being emitted and therefore, introduce an infectivity ratio ($r_{inf}$) between RNA copies and plaque forming units (PFUs). The complete conversion process (number of particles to RNA copies to TCID_50_) to estimate the viral emission rate is summarized by Equations (2)–(4) in Figure A1. This study makes the assumption that the concentration of RNA copies in each pre-evaporation particle is equal to the viral load measured by PCR tests. As the particle dries after emission from the respiratory tract, this viral concentration will increase with the decrease in particle volume.

Viral load measured by PCR tests was also found to vary considerably from subject to subject. For example, one study found that viral loads for SARS-CoV-2 positive individuals vary from 6.99 × 10^2^ to 4.71 × 10^8^ RNA copies/mL with a median of 1.46 × 10^5^ RNA copies/mL [74]. Viral load also depends on the stage of infection and the sample collection site [41,63,75,76]. In addition, a specific variant of SARS-CoV-2 may have an impact on the measured viral load [77].

### Appendix A.2. Particle Transport

The particle transport submodel describes the fate of particles emitted by the infectious individual in the surrounding environment. The near field transport and dispersion of respiratory particles takes place near the infected individual and within the first seconds of emission; it is dominated by effects of the initial jet flow produced by air escaping the mouth or nose, rapid evaporation as the particles enters a dryer environment, and rapid settling of larger particles. The evaporation and sedimentation of respiratory particles can be expressed as a function of time after emission from the infected individual [48]; these model results can be used to characterize the near field environmental fate of respiratory particles. Using this information for both saliva particles (oral generation) and lung fluid particles (bronchiolar generation), we can determine the size change and sedimentation of particles after the initial expulsion time period. Our implementation of this model did not account for near field dynamics except for estimating a final particle diameter after dehydration. Our model, therefore, assumes that particles quickly dehydrate to a stable particle size and are introduced into the well-mixed indoor environment; this assumption was partly due to our primary mode of modeling a steady-state concentration to model two individuals who are separated by over 2 m. The additional model and computational complexity from adding a complete characterization of near-field phenomena (e.g, jet flow emission) would be unnecessary for our numerical analyses and sensitivity analysis. If we were examining near-field exposures, including these dynamics would be necessary.

The far field transport and dispersion of respiratory particles takes place after most large particles have settled, the jet flow no longer impacts particle trajectory, and particles have reached a stable size after evaporative drying. For SARS-CoV-2, a time period of 50 s was chosen as the cut-off between near and far field transport models due to the fact that most particles have evaporated down to a stable particle size by that time or had already settled to the ground for those particles large enough to still be experiencing evaporation; note that because of the different time scales in which evaporation-induced particle size reduction and exposure occur (i.e., seconds v. hours) near field dynamics are captured in workflow of the particle generation submodel (see Figure A1 $d_{i}^{pre}$ and $d_{i}$ are used to distinguish between the pre-evaporation and post-evaporation particle diameters; moreover, the pre-evaporation particle diameter is proportionally reduced by a factor o*f* $r_{evap}$ as shown in Equation (1) of Figure A1.

Indoor transport and dispersion models include several mechanisms that reduce the concentration of airborne respiratory particles and airborne contagions. First, gravitational settling is modeled using Stokes law to determine the particle settling velocity for a given diameter $d_{i}$ (see Equation (5) in Figure A3); this settling velocity is used to determine a loss rate based on a conservative estimated initial particles height of 2 m above the floor of the room.

The second mechanism pertains to viral degradation in the surrounding environment. One study developed an equation to estimate a first order viral infectivity reduction rate for SARS-CoV-2 as a function of air temperature, humidity, and UV radiation [43]:

$$\begin{array}{cc} k_{inf}=0.16030 & +\;0.04018\left(\frac{\left(T-20.615\right)}{10.585}\right)+0.02176\left(\frac{\left(RH-45.235\right)}{28.665}\right)\\ \; & +\;0.14369\left(\frac{\left(S-0.95\right)}{0.95}\right)\\ \; & +\;0.02636\left(\frac{\left(T-20.615\right)}{10.585}\right)\left(\frac{\left(S-0.95\right)}{0.95}\right) \end{array}$$

where $k_{inf}$ is the first order viral decay rate (in min^−1^), T is the air temperature (in °C), RH is the relative humidity (in %), and S is the surface UVB irradiance (in W/m^2^). Finally, in the indoor setting, room air ventilation is also used to remove airborne particles from the air while replacing with clean air with zero particle concentration.

To account for these three far field mechanisms in the submodel, we adopt the approach of Dols et al., which uses an ordinary differential equation (see Equation (6) in Figure A3) to describe how the viral concentration changes with time [62]. The first term of on the right-hand side of Equation (6) is the time-dependent emission rate, which is determined by the viral emission rate defined in the preceding subsection. The summation of Q’s captures the effects due to indoor ventilation, whereas the penultimate term accounts for removal of virus via settling. The last term with $k_{inf}$ describes viral decay due to environmental factors. We note that this ODE has an analytical solution given by Equation (7).

### Appendix A.3. Human Response

To determine likelihood of infection from a contaminated exposure environment, the human response submodel first determines the inhaled viral concentration and then the subsequent total deposited dose. Figure A4 summarizes this workflow. Ultimately, this submodel aims to quantify the impact of infection.

The amount of inhaled virus is computed by multiplying the time dependent viral concentration for a given particle diameter with an inhalable fraction, $\theta_{i}$. One study presented an inhalability model that accounts for wind speed, minute ventilation and particle diameter [57]. Adopting the approach of [57], the particle diameter-dependent inhalability fraction is computed according to Equation (10) in Figure A4. For the indoor environment, a value of 0 m/s can be assumed for wind speed, resulting in an inhalable fraction of ~0 for 100-micron particles.

To determine likelihood of infection from an exposure environment, the model first accounts for how many particles are inhaled, the location of deposition, and the particle composition; this is determined by considering particle-size dependent inhalability [57], particle size-dependent deposition site [78] (which would also account for hygroscopic growth of a particle as it enters the humid respiratory tract [20]), and the viral content of particles [70]. The severity and course of illness of the disease then could depend solely on demographic factors, immune response, deposited dose, or a combination of factors. For SARS-CoV-2, experimenters were able to show clear dose-dependence for symptomatic/asymptomatic presentation of disease [17] as well as demographic differences in severity for symptomatic diseases [51,79].

An additional factor to consider is that of hygroscopic growth in the respiratory tract. The URT and proximal lung airways rapidly condition air to ~100% humidity and ~98.6 degrees Fahrenheit [80]; this environment will cause a hygroscopic particle to grow, often resulting in greater deposition in the URT and lower deposition in the LRT [20,24] (due to filtration by the URT).

Using a URT model that accounts for hygroscopic growth, we developed look-up-tables that accounted for ambient temperature and humidity, initial particle size, and initial particle composition for calculating URT and LRT deposition. Plots of URT and LRT deposition fractions as a function of initial particle size are shown in Figure A5.

A dose response model is typically used to determine the probability of infection, $P_{infect}$. Adopting the standard form of a probit model (see Equation (14) in Figure A4), $P_{infect}$ is taken to be a function of the total deposited dose $D_{total}$ (in virions, TCID_50_, PFUs, etc.), the median infectious dose $ID_{50}$ (in equivalent units), and probit slope β; note that here, $D_{total}$ and $ID_{50}$ are measured in terms of TCID_50_.

A model that can incorporate infectivity parameters (ID_50_ and probit slope) for both the URT and LRT is preferred when data exists to support such a model. If data supports site-specific infectivity, an ID_50_ and β will be available for both the URT and LRT. Although a study must be conducted to correlate infectivity likelihood with deposition site for SARS-CoV-2, a decreased infectivity from particles deposited in the URT when compared to the LRT has been observed for several biological agents including F. tularensis [81], Y. pestis [82] and B. anthracis [83,84]. It is possible to develop a joint probit model given the data or to simply assume that the probabilities of infection for the two deposition sites are independent by using:

$$P_{total}=1-\left(1-P_{URT}\right)\left(1-P_{LRT}\right)$$

where *P_total_* is the overall probability of infection, *P_URT_* is the probability of infection from the dose deposited in the URT, and *P_LRT_* is the probability of infection from the dose deposited in the LRT.

There is not sufficient evidence to correlate initial dose to disease severity or lethality in biological agents; however, for SARS-CoV-2 it has been shown that there are different infectivity parameters for asymptomatic and symptomatic presentation of the disease [85]. For SARS-CoV-2 we developed a non-dose dependent severity model based on documented incidence of severity, but it does not fit in the discussion of an end-to-end framework as it is independent of all other submodels. It is the authors’ belief that a model of viral reproduction and immune response is necessary for incorporating a model of disease severity within this framework.

## Appendix B. Results for First-Order Sensitivity

The first-order sensitivity indices produced by eFAST for the median and 95th percentile of $P_{infect}$ resulting from exposure to SARS-CoV-2 contaminated particles emitted by an infectious person breathing and coughing are presented in Figure A6 and Figure A7, respectively. The parameter rankings are displayed in Table A3.

**Table A3:** Ranking of the first-order sensitivity indices for the median and 95th percentile $P_{infect}$ with breathing and coughing as the emission mechanisms. Yellow shaded cells indicate parameters that were determined to be statistically significant.

Parameter	Parameter Description	First-Order Sensitivity Index Ranking			
		$\mathrm{Median}\;P_{infect}$		$95\mathrm{th}\;\mathrm{Percentile}\;P_{infect}$	
		Breathing	Coughing	Breathing	Coughing
$\varepsilon_{dz}$	Respiratory minute ventilation of the infectious person	4	6	6	9
$r_{evap}$	Ratio of evaporated particle size to initial particle size	7	4	8	7
$k_{inf}$	Viral decay rate	10	9	10	10
$\mu_{VL}$	Median viral load	1	1	1	1
$\sigma_{VL}$	Standard deviation of the viral load for the infectious individual	9	10	4	6
λ	Super spreader emission factor	8	8	5	5
$r_{inf}$	Infectivity ratio	2	2	2	2
$\varepsilon_{ndz}$	Respiratory minute ventilation of the SARS-CoV-2 susceptible person	6	5	7	4
$ID_{50}$	Median infectious dose	3	3	3	3
β	Base-10 probit slope for the probability of infection	5	7	9	8

## Appendix C. Python Implementation of End-to-End Modeling Framework

Python code for the end-to-end model is provided as supplementary material to this paper.
