# Supplementary figures and images for: A Novel Framework for Modeling Person-to-Person Transmission of Respiratory Diseases
Source: Viruses. 2022 Jul 19;14(7):1567. doi: 10.3390/v14071567 (PMC9322782; doi:10.3390/v14071567)

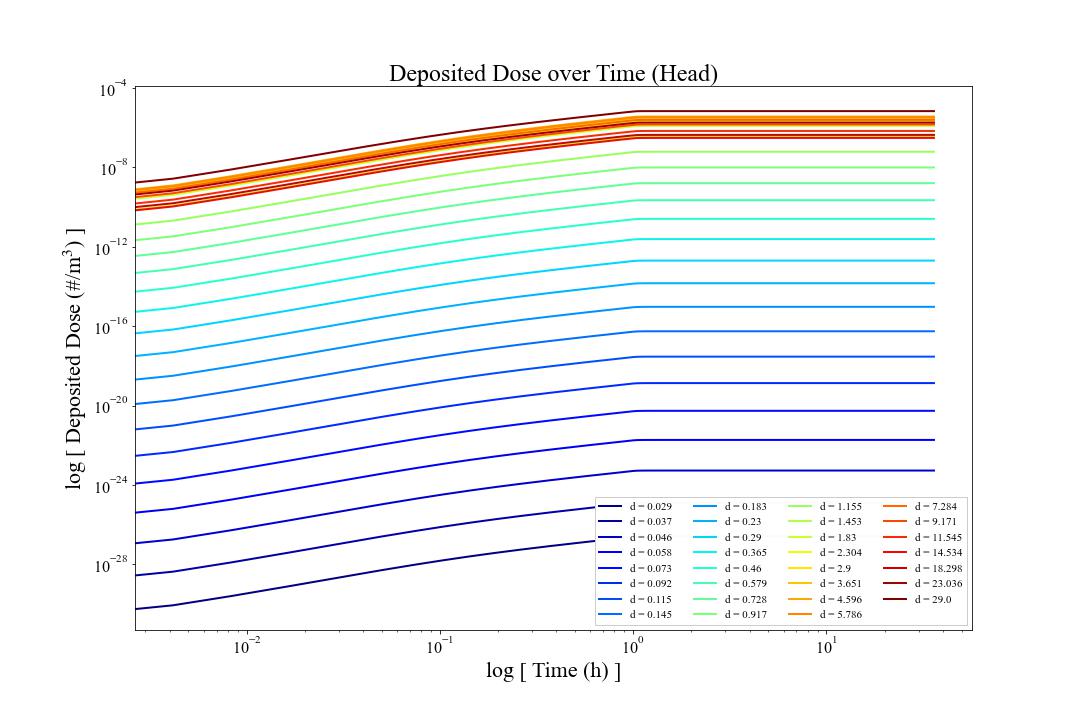

Supplement: Supplementary file 1 [file viruses-14-01567-s001.zip › End2End_COVID19_Python/Results/2022-07-20_coughing/Figures/depositedDoseHead_median.jpg]

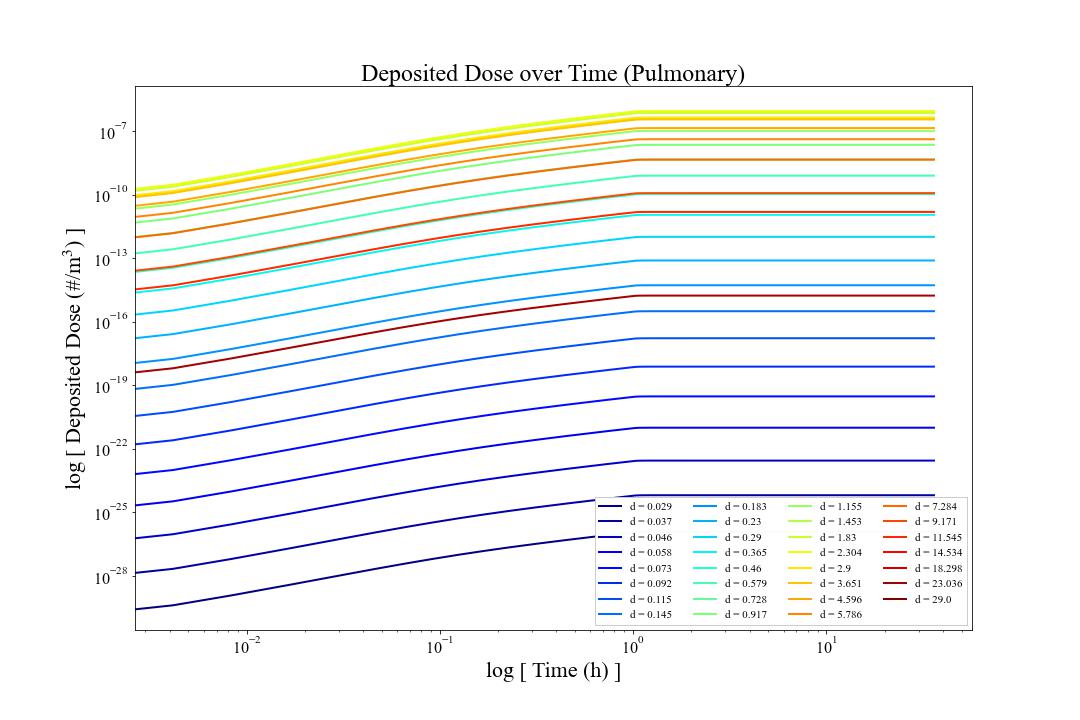

Supplement: Supplementary file 1 [file viruses-14-01567-s001.zip › End2End_COVID19_Python/Results/2022-07-20_coughing/Figures/depositedDosePulmonary_median.jpg]

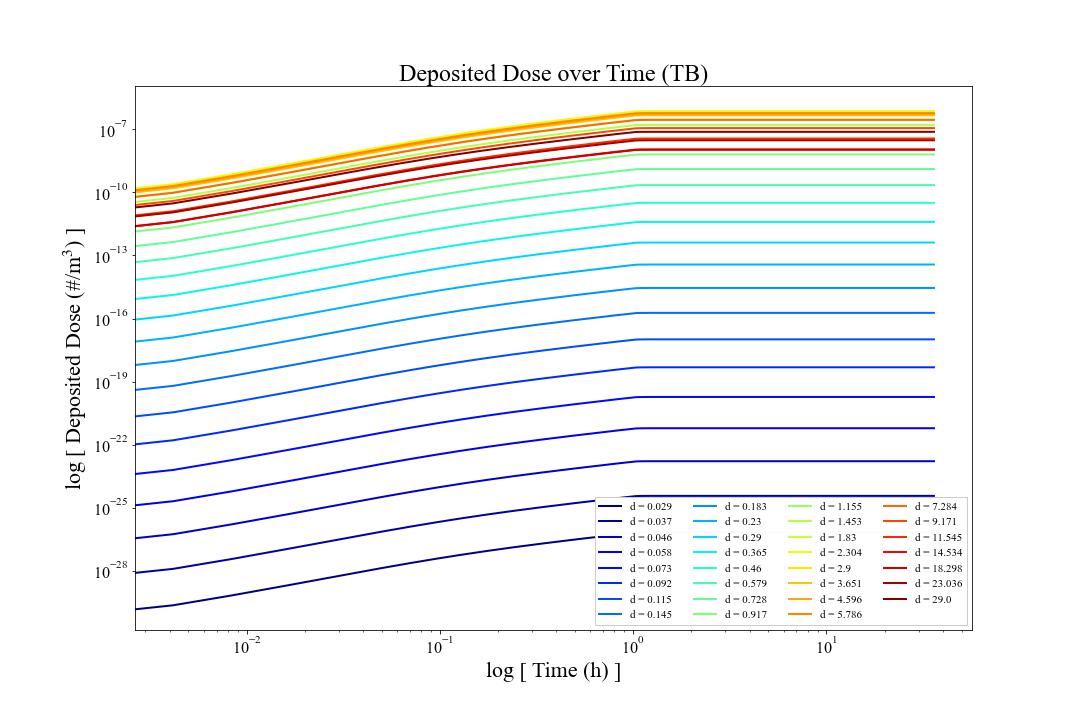

Supplement: Supplementary file 1 [file viruses-14-01567-s001.zip › End2End_COVID19_Python/Results/2022-07-20_coughing/Figures/depositedDoseTB_median.jpg]

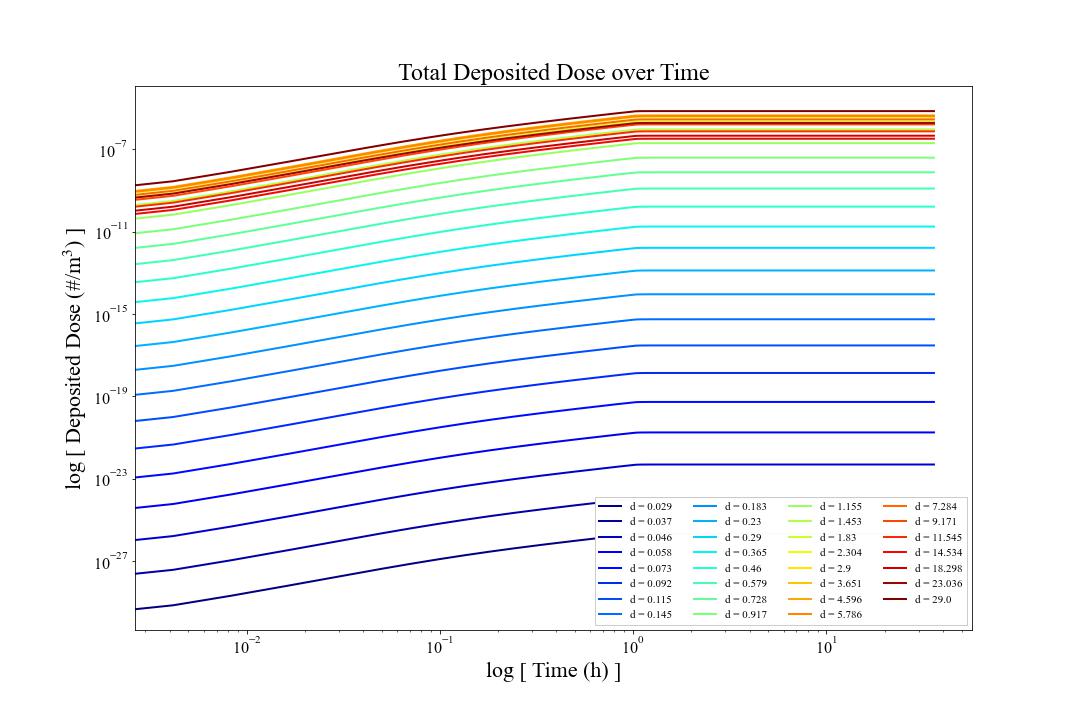

Supplement: Supplementary file 1 [file viruses-14-01567-s001.zip › End2End_COVID19_Python/Results/2022-07-20_coughing/Figures/depositedDoseTotal_median.jpg]

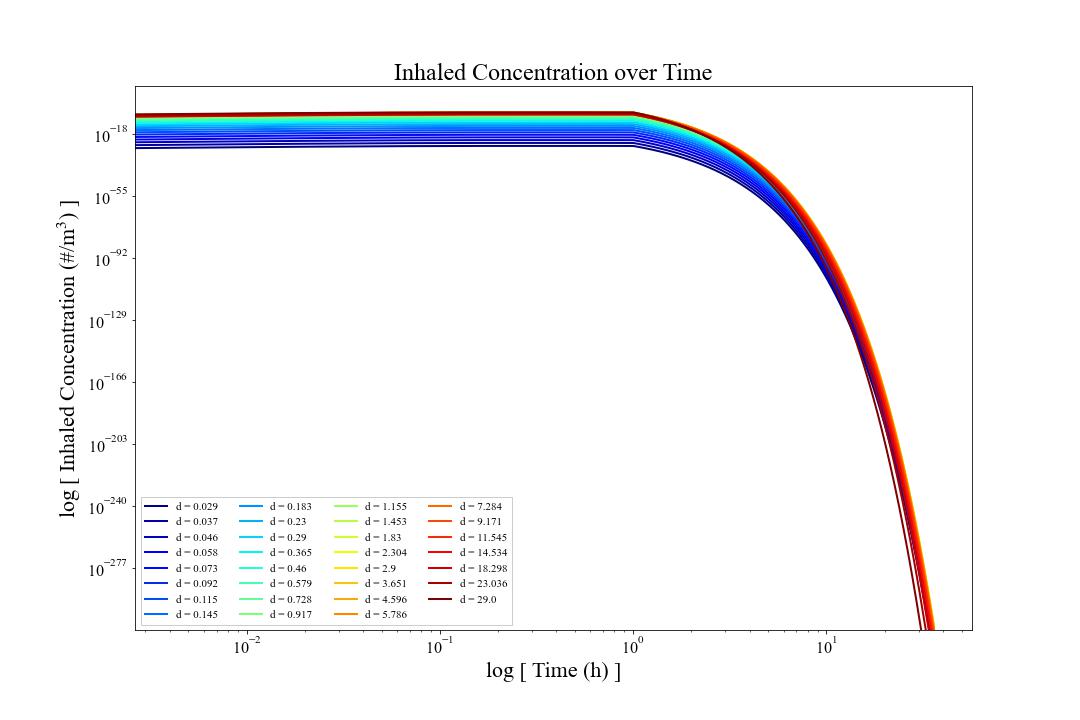

Supplement: Supplementary file 1 [file viruses-14-01567-s001.zip › End2End_COVID19_Python/Results/2022-07-20_coughing/Figures/inhaledConc_median.jpg]

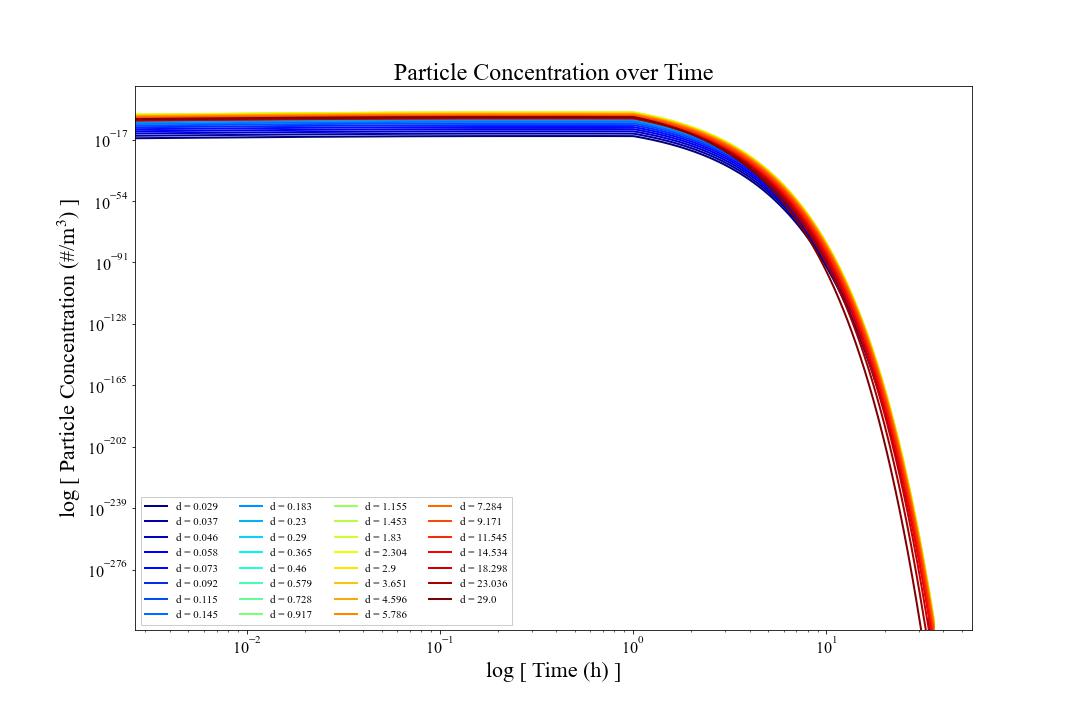

Supplement: Supplementary file 1 [file viruses-14-01567-s001.zip › End2End_COVID19_Python/Results/2022-07-20_coughing/Figures/particleData.jpg]

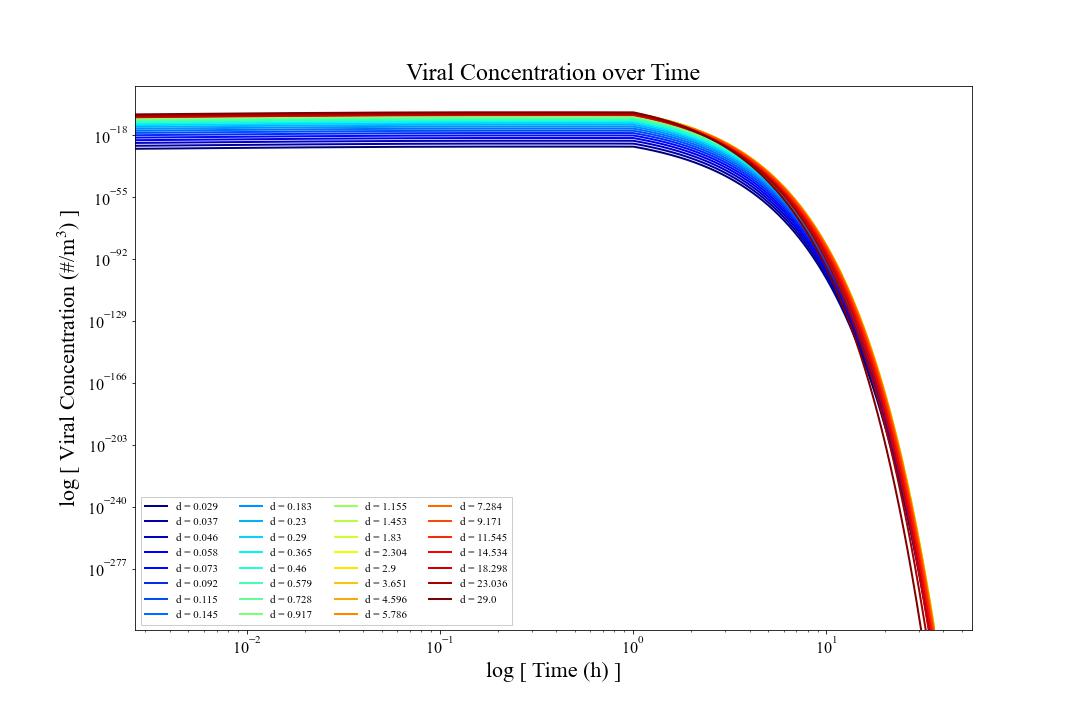

Supplement: Supplementary file 1 [file viruses-14-01567-s001.zip › End2End_COVID19_Python/Results/2022-07-20_coughing/Figures/viralConc_median.jpg]
